# Remote consultations in primary care: Patient experiences and suggestions for improvement

**DOI:** 10.1177/13591053241240383

**Published:** 2024-04-06

**Authors:** Richard O de Visser, Chimela Nwamba, Eve Brearley, Vahid Shafiei, Lia Hart

**Affiliations:** Brighton & Sussex Medical School, UK

**Keywords:** continuity of care, doctor-patient communication, patient satisfaction, qualitative method, remote consultations

## Abstract

The use of Remote Consultations (RCs) in primary care expanded rapidly during the Covid-19 pandemic: their ongoing use highlights a need to improve experiences of them. We interviewed 17 adults in the UK, including a sub-sample of five people with a First Language other than English (FLotE). Interpretative Phenomenological Analysis identified five major themes: (1) RCs are convenient, but they require appropriate technology and appropriate conditions of use; (2) even those with good general eHealth literacy and connectivity may struggle with systems that are not user-friendly; (3) greater reliance on verbal communication was experience as limiting empathy, and also made RCs more difficult for people with a FLotE; (4) RCs are considered inappropriate for complex conditions, or those with major psychological components; (5) continuity of care is important, but is often lacking. Overall, interviewees emphasised the need for more user-friendly processes, and greater attention to patients’ preferences for consultation type.

In-person consultations have always been part of the patient experience, but there is increasing use of remote consultations (RCs: also called e-consultation or telemedicine) along with increasing use of chatbots and other forms of artificial intelligence in healthcare. The UK National Health Service (NHS) published a Long-Term Plan in 2019 that proposed digital-first primary care, which would entail giving patients the choice of quick initial telephone or online consultations with general practitioners (GPs), with the aim of reducing time and costs associated with travel to, and provision of, in-person consultations (IPCs) (National Health Service [NHS], 2019).

Research conducted prior to the Covid-19 pandemic indicated that most patients would be willing to use RCs for advice on minor ailments or for ongoing conditions, and that many would also be willing to do so for emergency medical advice (Castle-Clarke, 2018). The speed of the switch to more RCs was accelerated during the Covid-19 pandemic. Although RCs were considered suitable in many situations, the key driver for the wholescale shift to this mode of delivery was the need for social distancing (Murphy et al., 2021). Patients generally responded positively to this: for example, in one study more than two-thirds of patients were ‘comfortable’ or ‘very comfortable’ attending RCs, and over three-quarters of GPs felt that greater use of RCs should be retained in the long-term (QualityWatch, 2023). However, it had been noted that RCs may demand specific skills and confidence in GPs that may require attention in training (Murphy et al., 2021), and it could be argued that RCs may also demand specific skills and confidence in patients, and that greater reliance on verbal communication may make RCs more difficult for people from linguistic minorities, as well as for people with impaired capacity for communication.

Patients, health professionals, and administrative staff recognise the potential value of RCs. They can enhance efficiency and reduce costs for practices: this is relevant at a time when GPs are finding themselves under increasing strain due to a rising workload and shrinking GP workforce (British Medical Association, 2023; Marchand and Peckham, 2017). However, because they provide a different mode of communication than IPCs, RCs may result in a different quality of communication, which could affect health outcomes (De Vries et al., 2014; Henry et al., 2012; Priebe et al., 2020). Verbal behaviours may not be greatly affected in RCs, if the audio technology is of sufficient quality. However, non-verbal behaviours are also an important part of doctor-patient exchanges: they can help to develop rapport, and displays of active or attentive listening are important for demonstrating empathy (Neighbour, 1987; Silverman et al., 2013). Non-verbal communication may be more difficult in RCs: there is no visual contact during telephone consultations, and during video consultations, non-verbal exchanges are limited to what can be shared via specific configurations of software and hardware.

Despite concerns about limits to communication, a recent review revealed that RCs are as effective and satisfactory as IPCs (Carrillo de Albornoz et al., 2022). Nevertheless, there are concerns about the ‘digital divide’ caused by disparities in access to, and comfort using, information technology. Engagement with RCs requires ‘eHealth literacy’: the ability to work with technology, and to engage meaningfully with professionals in electronic contexts (Norman and Skinner, 2006). Connectivity and eHealth literacy may be lower among older people, people of lower socioeconomic status, and ethnic and/or linguistic minorities (Centre for Better Aging and Citizens Online, 2021; Estrela et al., 2023; Saeed and Masters, 2021; Turner et al., 2022; Zibrik et al., 2015). However, the ability to engage with RCs is influenced not only by quality of the technology to which people have access, or their confidence in using it, but also the conditions of use, including whether people have the time and privacy needed, and how easily RCs can be accommodated within daily life (Grīnfelde, 2022; Norman and Skinner, 2006; Walthall et al., 2022).

It is important to explore the experiences of people accessing primary care via RCs since the end of Covid-19 restrictions and the establishment of a ‘new normal’ in primary care. This study was designed to examine the experiences of UK-based patients who have engaged in RCs with GPs. The aims were: (1) to gain deeper understanding of, and insight into, patients’ experiences of RCs; (2) to explore beliefs about how to improve RCs; and (3) to understand when patients feel RCs would be appropriate. With its focus on experience and meaning-making, Interpretative Phenomenological Analysis (IPA) was an appropriate method for addressing these aims (Smith et al., 2021). Although we applied the process of IPA and its primary focus on subjective experience, we were also open to interviewees’ beliefs about how their experiences compared to others’.

## Methods

### Sample

To ensure diversity across ages, purposive sampling was conducted with the aim of recruiting at least four interviews in each of four groups – women aged under 30, men aged under 30, women aged 40+, and men aged 40+– however, the self-selecting sample included three people in their 30s. Sampling was also designed to include at least four interviews with a first language other than English (FLotE), and the final sample included five FLotE interviewees. Requests for participants were shared with employers local to the host institution, and in various online fora. Further snowball sampling from interviewer contacts was also employed. IPA typically uses small homogeneous samples of 4–6 people (Smith et al., 2021). Larger IPA studies do not simply increase the sample size, but instead increase the number of homogeneous groups (e.g. the gender-age groups used here). This can allow greater breadth of coverage of social groups, but often entails sacrificing some idiographic focus. Participants were required to have experience of both IPCs and RCs. The 17 interviews were conducted by four of the authors: one conducted all FLotE interviews. Tags attached to quotes indicate the interviewee’s pseudonym, gender, age group, and FLotE background – for example, [Elizabeth: F60s FLotE]’ was a woman in her 60s with a non-English-speaking background. Access to raw data can be discussed with the corresponding author.

### Interviews

Interviews were conducted online in English. Informed consent was obtained online using procedures approved by the authors’ institutional review board. To build rapport and establish context for the discussion, participants were asked to describe their general health and their most recent RC experiences. The interview guide went on to cover five broad topics that reflected the study aims: (1) Feelings during RCs compared to IPCs; (2) Positive aspects of RCs; (3) Negative aspects of RCs; (4) Preferences for IPC or RCs in different scenarios; (5) Suggested improvements to RCs. Interviews primarily focussed on personal experiences, but respondents could also express their opinions about the possible experiences of others – for example, younger people discussed how older people may experience RCs.

### Analysis

Following IPA principles and procedures, analysis was conducted on a case-by-case basis before comparisons were made across age, gender, and language strata (Smith et al., 2021). Each transcript was hand coded with initial notes made to highlight topic-relevant phenomena: this was the phenomenological ‘P’ element of IPA and focussed solely on the participant’s account. To begin the interpretative ‘I’ element of IPA, the transcript was re-read, and each note was assigned a label denoting the emergent theme. Theme names and relevant transcript lines were then entered into a matrix to enable further analysis.

To establish a consistent approach to analysing the transcripts, the first author worked in pairs with each of four co-authors on their interviews. Within each pair, the interviewer and the first author independently conducted the phenomenological ‘P’ phase of IPA with one transcript, and then discussed common and divergent codes. Both members of the pair then independently conducted the interpretative ‘I’ phase, and then checked the correspondence of themes. Similar checks were used for subsequent transcripts, with the first author comparing the interviewer’s themes to their own reading of the transcript. There was no numerical assessment of agreement, as the focus was more on the iterative process of analysing each transcript rather than quantifying concordance within initial themes (Smith et al., 2021). When each transcript had been ideographically coded and analysed, comparisons were made across all transcripts, and themes and theme names used by the interviewers/analysts were compared, revised as necessary, and consolidated.

Each of the four interviewer-researchers analysed their own interviews to identify major themes and their subthemes. The lead author then combined, consolidated, and refined these themes, and returned them to all authors for verification. This process produced five themes which were then assessed for variation across age, gender, and language strata. This consolidation included applying agreed names to themes that may have been labelled differently by different analysts. As in many other IPA studies, it was not possible or appropriate to combine or merge themes that were not prevalent across all four sub-samples. Although there were no obvious differences between the four groups, as noted below, some themes were discussed differently by FLotE interviewees. The use of a larger sample increased the breadth of coverage of social groups, but word limits meant that less attention was able to be given to each individual and each quote.

## Results

Overall, patients reported many positive experiences of RCs and understood their value, but felt that they are not suitable in all contexts. They also felt that it was more difficult to establish rapport, and expressed concerns about a lack of continuity of care. Whereas the five major themes are presented here as distinct entities, there was some overlap: a point returned to in the discussion. The key elements of each theme are described below and illustrated with verbatim quotes. This includes discussion of elements for which there was ambivalence, or that were positive and negative like two sides of a coin – for example, information technology was easy to use and efficient as long as people had good access to equipment and good connectivity. In many cases, respondents gave positive accounts of their own experiences, but also expressed an awareness that others’ experiences may not be so positive.

### Theme 1 convenience and comfort, confidence and confidentiality

Interviewees generally had positive opinions of the accessibility of RCs, but also noted important personal and contextual factors that affected their experiences.

#### Convenience and comfort

Many interviewees described RCs as more convenient than IPCs. For example, David **[**M20s**]** noted that RCs are ‘more accessible and comfortable’, and Bob described how RCs are satisfying because they remove many of the logistical and organisational barriers that accompany IPCs:

*It would have meant a trip into [Location], and all the struggle and grief of the parking […] It would have meant quite a journey. But really what we wanted was immediacy of feedback and getting on to the next part of the diagnosis. [Bob: M50s]*

Interviewees also appreciated that RCs enabled them to obtain medical care if they were not well enough to travel. For example, Keith [M 60s] appreciated that RCs allowed communication with a health professional even when illness meant that he felt that he was ‘not up to the journey’ to the clinic. Interviewees also noted that not attending in-person meant that they were not exposed to others’ infections. In addition, interviewees expressed awareness that many people might find RCs more accessible for various reasons including distance, restricted mobility, and caring responsibilities:

*I’m sure for lots of people who live in more remote areas or are less able-bodied or less able to sort of freely travel, it gives them access to care maybe in a way that they wouldn’t you know- wouldn’t be possible otherwise […] If you are able to just make a phone call or have someone call you, it’s convenient. I guess that also applies if you’ve got young children or you are a carer or something like that which might also stop you from being able to go to a clinic or to a GP. [Alice: F20s]*

Some interviewees also noted that RCs may also be more convenient for health professionals. Jamal [M60s FLotE] noted ‘it saves a lot of my time and doctors’ maybe time’, and Molly noted that the greater convenience and efficiency of RCs could benefit doctors, and therefore lead to more satisfying care for patients:

*It’s better time-wise and some GPs can do it from home. It probably increases the number of appointments that GPs can offer, which means more patients will be satisfied with the service, which is good. [Molly: F30s]*

Interviewees also noted that some elements of the RC process could be completed outside of typical working hours. For example, online triage forms could be completed at any time and in any place, and this obviated the need to call early in the working day with the hope of booking one of the finite number of consultations. Some interviews noted that the process worked best when they had advanced warning of when they might be contacted for RCs. For example, Faith [F20s FLotE] noted that knowing when to expect a callback allowed her to ‘prepare yourself and put yourself in a place where you can have the consultation’, and Elizabeth explained how advanced warning allowed her to create privacy in a public space that allowed her to prepare herself so that she could engage fully with the consultation:

*Just as soon as I arrived to work, I said to my colleagues said ‘OK, I need 10 minutes. I need a short time talking with the doctor from this time to that time’, and they said, ‘It’s OK’. I prepared them and I was prepared myself. [Elizabeth: F60s FLotE]*

#### Confidence and confidentiality

An additional benefit noted by many interviewees was that RCs provided distance from the GP that made them more willing to discuss sensitive or private topics. Alice [F20s] noted that for people who lack confidence ‘it might be easier to do online calls or phone calls’. Indeed, Elizabeth noted that the physical and interpersonal distance that characterises RCs alleviated her shyness, and made her feel better able to express her concerns, with the implication that this would lead to better healthcare experiences:

*I have to be honest, I’m a little bit shy person. I find that on the phone I can talk better and explain better my problem rather than face to face see the doctor. [Elizabeth: F60s FLotE]*

Others suggested that simply being in the familiar and comfortable context of their own home made them more willing and able to discuss sensitive or private topics. As suggested by Elizabeth, Jamie indicated that better healthcare experiences and outcomes may result from being more comfortable in his own home and therefore more willing to discuss sensitive issues:

*I believe I have more confidence online, as you are in the comfort of your own home rather than a doctor’s office, and more willing to talk about conditions and personal issues.* [*Jamie: M20s]*

However, some interviewees noted that not all living arrangements provide the privacy required for detailed, candid and confident discussion. For example, Keith [M60s] suggested that it is ‘unseeming to talk about poo problems with others that can overhear’, and Alice [F20s] noted that in some cases it may not be safe to discuss health concerns in the presence of other people. Molly [F30s] described an experience that may be familiar to the many younger adults who live with their parents. She described apprehension about being overheard by other members of her household when discussing sensitive issues. This discomfort must be balanced against the convenience referred to above:

*With mental health issues I wouldn’t feel comfortable because someone in my home may hear me speaking or at the practice. For most issues I think it’s fine, but for some they definitely need to be seen in person especially for some people. I’ve felt a few times that I didn’t want a phone conversation at my parents’ house, so I’ve actually had to leave the house if it’s an uncomfortable consultation. [Molly: F30s]*

### Theme 2 technology, usability, and digital literacy

There was a duality related to interviewees’ perceptions of technological aspects of RCs. Interviewees described their own experiences as relatively trouble-free, but they often presented this in relation to their knowledge or perceptions of others’ experiences. They appeared to be aware of the ‘digital divide’, which meant that not everyone would have access to the technology or connectivity required for RCs, or feel comfortable and confident using it. For example, Molly linked her perception of the ease of RCs to her age-related competence and confidence with communication technology, but contrasted this with people from older generations:

*As I am young, I found it easy, but for my mum who isn’t tech savvy, I think she would struggle. People good with technology would be fine with it, but for example, a 75-year-old would struggle. [Molly: F30s]*

However, even younger adults whose jobs involved the use of IT noted that not all aspects of the RC process were easy to navigate. For example, John suggested that older people and/or those with neurodegenerative conditions might have difficulties with technology and that this could add to their distress, but he described how he had also felt frustrated by some elements of the process himself, and that he had needed help to complete them:

*People who, you know, might not have very much money and – or who aren’t very tech savvy … I can imagine them experiencing it as quite stressful uploading pictures, you know? My dad’s 76. He’s got Alzheimer’s. If they said to him upload pictures, he wouldn’t have a clue what to do and it would cause him a lot of distress. […] It’s really interesting you ask me that. Yes, I asked somebody else to help me with it, because I’m not very tech savvy and I get frustrated. [John: M40s]*

### Theme 3 doctor-patient communication

Interviewees generally reported positive experiences of doctor-patient communication in RCs, but there were some notable caveats. First, interviewees noted that because visual cues were reduced (in online RCs) or absent (in phone RCs), there was a greater need for high-quality verbal communication. Second, following on from this, FLotE interviewees noted that they found communication in RCs more difficult than in-person.

#### Information exchange and empathy

A unanimously identified limitation of RCs is that they provide less visual information than IPCs. Interviewees highlighted the importance of IPCs to fully inform health professionals. Chantelle felt that physical proximity and contact were needed to conduct the thorough assessment necessary for proper diagnosis of the nature and severity of a condition – for her touching and feeling appeared to lead to better understanding:

*He can’t even, you know, assess you physically … The patient needs to be touched; you need to feel the patient. You need to understand where this pain is. You need to do a physical assessment to assess the severity of the pain. [Chantelle: F50s]*

However, perhaps more important for patient experience was the feeling that RCs are ‘less human’ [Alice [F20s]. Keith [M60s] was typical of many interviewees in noting that although RCs obviously involve some kind of interpersonal communication, he was disappointed that ‘there is no personal interaction’. Most interviewees referred to their own preferences for in-person contact and/or their perceptions of whether healthcare professionals were as engaged in RCs as they were in IPCs. For example, John noted that RCs affected how he felt, in relation both to his own behaviour and to his perception of whether the health professional’s behaviour indicated that they were taking his concerns seriously:

*I am just not a phone person. I am not natural over the phone … I’m quite good at, you know, I’m quite a social person, so I’m quite good at responding to visual cues. […] Also you’re just not able to see what your GP is doing or saying or like how his body language is when you’re over the phone. So yeah, you don’t know if he is taking it as seriously as they could. [John: M40s]*

In contrast to John, David knew that his preference for RCs was non-normative, but he also explained his feeling that RCs may not provide the same quality and/or quantity of information as IPCs. He also implied that better doctor-patient relationships depend on doctors developing a broader and deeper understanding of the patient’s concerns:

*I don’t think that I’m in keeping with the normal population with this … I think I’m more willing to seek help online than I am in person. […] It’s much more difficult to get a concept of the patient as a whole – or me as a whole – the issue: how it’s impacting me, and I guess what I am truly concerned about […] If you were in person I feel like they would be able to get a better scope of how it’s impacting my life and probably form a better patient-doctor relationship. [David: M20s]*

Interviewees felt that it was harder for doctors to be empathic in RCs. For example, Faith [F20s FLotE] noted that ‘it’s difficult to show empathy through the phone’, and Carol [F50s] suggested that ‘just being face-to-face with someone is more reassuring’. Ami noted that the combination of in-person contact and continuity of care meant that health care professionals could develop and reflect a real sense of knowing each patient as a person with their own biography, and to understand a specific consultation in the context of an ongoing doctor-patient relationship:

*When I was in person he would say ‘You know, I noticed that you’re looking quite tired’… you know that sort of thing can’t be noticed when you’re over the phone or even online. [Ami: F20s]*

Bob expanded on these ideas by suggesting that not only were expressions of empathy important, but that entering the treatment setting is also important. He indicated that he felt less confident in his GP during a RC than during an IPC in what was obviously a medical context furnished with all of the medical equipment and resources that might be needed to provide thorough medical care. The IPC setting gave Bob a feeling of confidence in his doctor that was absent from RCs:

*You want confidence in the person that’s providing you advice on your care – and I think that’s probably easier to get that confidence if you’re in person and surrounded by, I guess, all of the paraphernalia at the surgery, whatever it might look like, in person, than you can online. [Bob: M50s]*

#### Barriers to communication

Although their experiences of RCs were generally positive, the FLotE interviewees noted that if they struggled to understand strong accents during in-person interactions, then this difficulty was exacerbated in the specific context of RCs. Interviewees noted that the lack of non-verbal cues made phone conversations more difficult than those involving a visual component. For example, Alex noted that phone consultations were difficult because he was unable to lip-read:

*The only difficulty for me comes when, as you know, English is not my first language, so I do struggle when I’m talking to doctors that also have strong accent for example. And for me part of communicating is reading their lips. So when I’m talking to someone and I’m seeing the face, it’s easier for me to understand them than if they have a strong accent over the phone. That I’m at a struggle to understand what they’re saying. [Alex: M30s FLotE]*

However, interviewees noted that if they did alert others to these difficulties, then it was possible for them to be addressed by slowing down their rate of speech, using more accessible language, and checking comprehension:

*Maybe they don’t understand me because my English is not very good, but actually, no. With them – and even I, for example, like now I said ‘No sorry. I don’t understand’. They tried to, I don’t know, talk slowly, or change their words, make sure that I understand, and they explain very well for me. [Elizabeth: F60s FLotE]*

### Theme 4 appropriate uses of RCs

Interviewees expressed clear preferences for when they felt RCs were appropriate and acceptable. They acknowledged that online form filling and RCs are an important part of triage procedures, and were in broad agreement that RCs were suitable if their medical concerns were ‘ordinary’ (Jamal (M60s FLotE)), ‘superficial’ (Carol (F50s)) ‘not severe’ (Andrew (M30s FLotE)), or ‘non-life-threatening’ (Alice (F20s)). However, they noted that RCs may not be sufficiently informative for ambiguous symptoms that are difficult to describe or locate, such as referred pain, or for complex or overlapping conditions:

*If they need examination, or maybe I can’t explain what’s really my problem. And sometimes you have got a pain somewhere, and actually the pain is actually from somewhere else. [Elizabeth: F60s FLotE]*

. .

*If someone doesn’t fully understand the problem or know where the problem is originating from, it’s harder to discriminate and sort of identify…if someone has a little bit like, you know, layered conditions, they don’t know what came first, They don’t know what problem is the biggest problem, and more what’s the most prominent issue? It’s a lot harder to have that talk over the telephone. [Faith: F20s FLotE]*

Faith went on to explain that if there was any ambiguity or lack of certainty, then IPCs would provide further opportunities for exploration by the doctor and explanation by the patient, noting that in-person ‘there’s a lot of cues that can be given’ compared to in RCs.

Following on from this, one must bear in mind that different types of RCs exist, with telephone RCs providing no visual or tactile information, and video RCs providing some visual information, but no tactile information. However, information exchange was not the sole factor to influence preferences: whereas some interviewees liked the anonymity of RCs, many responded negatively to the lack of personal contact for psychological concerns, or conditions that evoked strong emotions. Like many interviewees, Alex [M30s FLotE] noted that it in relation to mental health ‘I would prefer to do it in person than do it on the phone’, and he went on to explain that this was because ‘you can feel the better emotional connection, and emotion is what you_[sic]_ probably struggling with. Andrew said:

*I think [for psychological issues], there should be a level of empathy, perhaps would be better when it’s face to face it’s more, it’s difficult to say, it’s more natural. It’s a little bit like, I don’t know, breaking up with your girlfriend over text or in person, it’s impersonal. [Andrew: M30s FLotE]*

Similarly, Faith believed that strong and difficult emotions were best addressed in IPCs. Her repeated use of the words ‘whole’ and ‘interaction’ suggested that IPCs not only give more information, but also provide a better experienced of being attended to and cared for:

*I still feel like face to face is a lot better for mental health because of the whole people need to feel the interaction. I feel like the whole personal interaction adds to the reassurance that they are getting the help that they need, and they don’t feel like they’re being rushed or they don’t feel like certain things are being overlooked, They feel like they are physically being seen and the doctor’s giving them all that attention directly to them, whereas if they have it on a zoom call, they don’t have that connection and that interaction that they may be wanting. [Faith: F20s FLotE]*

Interviewees generally felt – regardless of whether the primary presenting concern was physical or psychological – that patients should be given options that take into consideration their capabilities and their preferences. The quote below suggests that the communication mode should be determined by the specific needs of a specific person in a specific context:

*Ultimately, I think there should be some face to face and some telephone appointments, or even video calls but they will have their own issues with people who can’t use technology very well or don’t have good Wi-Fi connection. [Molly: F30s]*

### Theme 5 continuity of care

Although it was noted earlier that interviewees considered RCs suitable follow-up for ongoing conditions, they also emphasised that continuity of care was important not only in this context, but more broadly. Many interviewees commented negatively about experiences of lack of a continuity that was not only inefficient, but which also eroded their sense of confidence and their feelings of being cared for:

*One problem is patients’ continuity. Who they speak to, perhaps, when they’ve got a call from a doctor. They necessarily don’t know who they’re talking to. Maybe again that information could be provided as well: Who is the doctor that they’re going to be talking to? And maybe a choice for the patient as well, which doctor they could choose, because if the patient [is] being seen by a particular doctor and then someone else comes and have a chat with them, it’s, you know, they may not know their background, and may not give the optimal treatment that they would have had. [Andrew: M30s FLotE]*

Interviewees also noted that RCs must be properly integrated within functioning, linked-up systems of care. Many interviewees reported experiences of a lack of follow-up. Others indicated that even when they did receive appropriate follow-up, there was a sense that it was easy to get (at least temporarily) lost or overlooked lost within busy and overworked systems. Many interviewees suggested that the increased and widespread use of remote consultations increased the risk of being lost within systems and/or of individuals having the required information but not having a sense of the whole person and their history. For example, Brian noted that the complete lack of in-person contact during Covid-19 restrictions meant that he did not know who his GP was. He added that a lack of repeated or ongoing contact with any single GP meant that he lacked confidence and trust in any of the GPs. He highlighted how continuity allows the development of rapport, trust, and feelings of being cared for:

*I don’t even know who my doctor is. I don’t know who I have. I don’t even have confidence in any of the doctors because I don’t know any of them very well. Because for you to have confidence and trust in someone, you should have seen them before and developed a rapport with the person and feel cared for by them. However, this is very difficult to achieve online, so it’s also difficult to open up more about your problems online. [Brian: M20s]*

According to some interviewees, not only did remote consultations involve less continuity and increase the risk of getting overlooked, but they also reduced their confidence in their ability to assert their desire for proper follow-up and continuity of care. The quote below illustrates that some patients felt both less visible and less assertive in RCs, and that this may be especially so for patients who are experiencing low mood or low motivation as part of a physical condition:

*Potentially I would have been more assertive had it been in person, um and maybe I would have been more likely to follow up on it, while with phone calls it felt like even though the phone calls themselves were positive, I didn’t take the name of the person I was speaking to or anything like that, so following it up felt more difficult, and especially at a time when I was quite low […] The phone calls themselves were positive and good, it’s just that the outcomes of the phone calls well, more that there wasn’t an outcome*

*Interviewer: Mm, so it’s the follow-up that was lacking?*

*Yeah, yeah exactly. I think the care I received during the phone calls was, you know it was to a high standard, and I felt like I was being listened to and like something was going to happen, but it feels like maybe you know it just got lost or something in the filing system [Alice: F20s]*

Chantelle [F50s] noted one instance of lack of follow-up or lack of continuity of care linked to technological difficulties, explaining that ‘the call cut off and I didn’t get any call back from the GP and I had to ring 111 because the GP was about to close’. However, other interviewees noted that remote consultations are by their very nature characterised by less continuity of care and more frequent shifts between health professionals. The quote below illustrates that such shifts can result in in patients not feeling that they and their concerns are fully known or fully understood:

**‘**I feel like [in-person] they are more likely to ask you, you know, follow up questions and really delve deeper into what any issues might be or like why the reason you’ve come here today. Umm … and also, they have a much better continuity of care, if that makes sense. So they’re much more likely to remember you from your previous consultations and, you know, ask about a specific follow up because they’re very aware of the situation. They know your story, if that makes sense’. [Amy: F20s]

This situation was perceived as worse when there were shifts between staff with different roles or professions. The quote below highlights that when patients expect to have a consultation with a doctor, then they should actually be seen by that person, rather than another staff member:

I called the GP and I told them I wanted to speak to the doctor regarding it [chest pain] and they actually told me a doctor was gonna call me and I was expecting a doctor to call me and someone did call me. She did not introduce herself. She just said a name was Maria. She was calling from the practice. And I was listening to what she had to say, and she was asking me so many random questions regarding my symptoms and all of that. I gave all the information to her only for me to find that at the end of the call that she is a nurse. I found that she was a nurse and I got disappointed because initially I was told the doctor was gonna call me [Chantelle: F50s]

These accounts suggest that continuity of care improves the quality of care because it increases feelings of trust and results in better exchanges of information between patients and HCPS.

## Discussion

The key finding of this study was that although patients saw value in RCs and had good experiences of them, they also noted that RCs are not suitable in all contexts, and felt that patients should have greater influence on when and how they are involved in ICPs or RCs. Even among people capable and confident enough to complete online interviews, there were many difficulties and barrier related to RCs. This suggest that the experiences discussed in this paper may be even more pronounced among people who were not able to take part in the study.

Although the themes were presented as distinct entities above – and will be below – there was considerable overlap between them. For example, the interpersonal aspects of doctor-patient communication (Theme 3) fed into experience of, and beliefs about appropriate uses of RCs (Theme 4) and were a component of continuity of care (Theme 5), and the quality of doctor communication was influenced by having a FLotE, and also whether the mode of RC allowed for any visual communication (as in video RCs) or none (as in telephone RCs).

In the first theme, patients highlighted the convenience and comfort afforded by RCs, and noted how this could increase their confidence in medical consultations. In line with the NHS digital first strategy, they noted that RCs could reduce waiting times and reduce the time and costs associated with travel to, and provision of, IPCs (NHS, 2019). Furthermore, recent reviews have revealed that RCs are as satisfactory and effective as IPCs (Carrillo de Albornoz et al., 2022). However, in concordance with past research, interviewees noted that the ability to engage with RCs is influenced not only by access to the relevant technology, but also the conditions of use, most notably privacy and confidentiality (Norman and Skinner, 2006; Walthall et al., 2022). Furthermore, other experiential studies have noted that the removal of transitional spaces and experience (e.g. time in a waiting room) mean that people may have to do more psychological and emotional work to prepare themselves for RCs and to re-adjust to everyday life afterwards (Grīnfelde, 2022).

The second theme addressed access to technology, digital literacy, and usability. As noted in the introduction engagement with RCs requires ‘eHealth literacy’, and it has been noted that connectivity and eHealth literacy may be lower among older people and people of lower socioeconomic status (Centre for Better Aging and Citizens Online, 2021; Saeed and Masters, 2021; Turner et al., 2022). However, interviewees’ accounts suggested that even those with good general eHealth literacy and connectivity may struggle with RC systems that are not user-friendly. There would therefore be value in exploring how to make all aspects of RCs easier for all patients.

In the third theme, it was noted that the quality of doctor-patient communication in RCs was generally considered satisfactory, but that greater reliance on verbal communication could make RCs more difficult for people with FLotE backgrounds and people with impaired capacity for communication. This could limit information exchange, and was also noted to affect the doctor-patient relationship and feelings of empathy. Interviewees also noted that beyond interpersonal elements such as empathy, the ritualistic elements of IPCs are missing from RCs. These aspects of IPCs have been shown to have a meaningful impact on patient experience and outcomes, but are necessarily absent from RCs (Benedetti and Amanzio, 2011; Costanzo and Verghese, 2018). Models of communication emphasise the importance of non-verbal behaviours which may be restricted by, or impossible in, RCs (Neighbour, 1987; Silverman et al., 2013). This may be most apparent in telephone RCs because there is a complete lack of visual communication; it may still be evident in video RCs where there is (limited) visual communication, but a lack of touch. Limitation to non-verbal communication in RCs were reported to restrict empathy and the establishment of rapport: this is important because perceptions of lower doctor empathy have been associated with poorer treatment adherence, and poorer outcomes (Derksen et al., 2013; Kim et al., 2004). The findings suggest that in RCs there may be a heightened need for GPs to communicate well and to actively explore – and respond empathically – to all patient concerns.

The material presented in the fourth theme revealed that interviewees were happy for RCs to be used for minor or non-urgent health concerns or for follow-up from earlier IPCs, but that they felt that they were not appropriate in urgent situations, for complex conditions, or for conditions that had a strong psychological component. To some extent, these findings reflected those of studies conducted prior to changes enforced by Covid-19-related social distancing (Castle-Clarke, 2018). Post-Covid-19 social distancing, a ‘new normal’ has been established with consolidation of the NHS long-term plan for digital-first primary care: however, whereas the NHS Long-Term Plan in 2019 proposed digital-first primary care, it also noted the importance of giving patients some choice in relation to the mode of communication for initial consultations (NHS, 2019). The importance of such patient choice has been noted in other qualitative studies of RCs (Kristiansen et al., 2023). Whereas some interviewees reporting feeling more willing or able to discuss sensitive issues in RCs, others preferred IPCs for such discussions. The lack of consensus around the preferred mode of consultation of sensitive topics suggests that it may be important to ask patients their preferred mode of discussion of specific topics rather than assuming that one mode is suitable for all patients or that one mode is always suitable with the same patient.

The fifth theme highlighted how important continuity of care was to interviewees. Continuity of care refers to repeated contact between a patient-doctor dyad, and it is important because it may foster better understanding of each other’s views and priorities (Gray et al., 2003), and it is also an important influence on patient satisfaction and health outcomes (Bazemore et al., 2023; Dyer et al., 2022; Pereira Gray et al., 2018). Concerns about a lack of continuity of care are not specific to RCs (Gray et al., 2003; Parry et al., 2023). However, given that many patients felt that it is more difficult to establish rapport in initial RCs than in IPCs, the issue of continuity in subsequent RCs may be more even more pressing. The findings suggested that the reduced personal interaction that characterises RCs makes continuity of care even more important in RCs than it usually is in IPCs for the establishment and maintenance of feelings of being cared for.

### Strengths, limitations, and suggestions for future research

A strength of this study was that the sample was designed to ensure diversity across age, gender, and language to enable comparison of experiences across these variables. There were no obvious gender differences. Although there were no obvious differences in the responses of older or younger participants, participants of all ages noted that people older than themselves may not be as confident with technological aspects of RCs. The inclusion of older interviewees (e.g. over 70) would have enabled exploration of whether the opinions of their younger counterparts were accurate. As noted in Theme 3, language was a clear influence on actual and potential experiences of RCs, with people from FLotE backgrounds noting that the greater reliance on verbal communication in RCs meant that they could be more challenging than IPCs. Because we were unable to include people who were unwilling or unable to complete an interview in English, we may have underestimated to impact of English language proficiency in RCs. Future research could usefully include interviews with people from various FLotE backgrounds conducted in their first language.

The planning and execution of the study were informed by recommendations for quality and validity in qualitative research (Malterud, 2001; Yardley, 2000). *Sensitivity to context* (Yardley, 2000) was addressed by identifying the personal, micro-social, and macro-social influences on interviewees’ experiences – for example, exploring the experience of ethnic/linguistic minorities, noting our own interests as health researchers, and acknowledging how the process of online interviewing may have affected discussion of online medical consultations. In relation to *interpretation and analysis* (Malterud, 2001), *transparency and coherence* (Yardley, 2000) were demonstrated by clearly explaining how themes were generated, and by using various quotes to show how themes were grounded in the data. *Commitment and rigour* (Yardley, 2000) were promoted by the authors independently coding transcripts before conferring to ensure a rigorous and consistent application of the IPA process. Reliability was ensured by regular communication within the four pairs of authors to resolve differences of perspectives on emerging themes. *Impact and importance* (Yardley, 2000) and *transferability* (Malterud, 2001) were demonstrated by showing how the results could inform better practice in RCs to improve healthcare interactions and health outcomes.

Future research could explore a broader range of characteristics that may affect engagement in, and satisfaction with RCs. For example, there would be value in including patients with visual or auditory impairments. In relation to accessibility, it must be noted that all interviewees were happy to complete interviews online, and this may have skewed the sample towards those more able to engage in RCs. The consequence of this may have been an underestimation of the difficulties some people have with RCs. Indeed, many participants noted that other people may have found RCs to be more challenging than they had. Further effort would be required to study the perspectives of people who are unable or unwilling to engage with RCs.

In addition to further exploring patient perspectives, there is a need to better understand the perspectives of service providers. Some studies have described health professionals’ opinions and experiences. For example, qualitative studies with physicians have highlighted their belief that RCs demand more effective communication skills and more active and focussed patient involvement (Björndell and Premberg, 2021). Furthermore, one UK study conducted prior to the Covid-19-inspired widespread application of RCs revealed that RCs were not perceived by practice staff to be creating sufficient efficiencies to warrant financial investment (Farr et al., 2018). The researchers who conducted that study suggested a need to better understand how patients believe the system should be used and how it can be improved. However, it is also important to better understand what administrative and managerial staff in primary care experience as the benefits and disadvantages of RCs, and how they can best balance what currently works for them, what needs to be improved for them, and how they can address patients’ needs.

## Conclusion

The three aims of this study were to explore patients’: (1) experiences of RCs; (2) beliefs about how to improve RCs; and (3) beliefs about when RCs are appropriate to use. The data revealed that patients generally had good experiences of RCs, and saw some value in them. However, they felt that health care professionals should attend more to patients’ preferred consultation mode in general, and for specific health concerns. Interviewees emphasised the need for more user-friendly processes, greater attention to patients’ preferences for consultation type, and better continuity of care.

## Supplemental Material

sj-docx-1-hpq-10.1177_13591053241240383 – Supplemental material for Remote consultations in primary care: Patient experiences and suggestions for improvement Supplemental material, sj-docx-1-hpq-10.1177_13591053241240383 for Remote consultations in primary care: Patient experiences and suggestions for improvement by Richard O de Visser, Chimela Nwamba, Eve Brearley, Vahid Shafiei and Lia Hart in Journal of Health Psychology
