# Supplementary material for: Remote consultations in primary care: Patient experiences and suggestions for improvement
Source: J Health Psychol. 2024 Apr 6;29(12):1321–35. doi: 10.1177/13591053241240383 (PMC11462776; doi:10.1177/13591053241240383)
Supplement: sj-docx-1-hpq-10.1177_13591053241240383 – Supplemental material for Remote consultations in primary care: Patient experiences and suggestions for improvement [file sj-docx-1-hpq-10.1177_13591053241240383.docx]

**Supplementary table**: Interviewee characteristics

| **Pseudonym** | **Gender** | **Age** | **Biographical sketch** |
| --- | --- | --- | --- |
| Alex | Male | 30s | Has chronic sinusitis and hay fever, and previously had surgery for removal of a nasal polyp. He Consults an ENT specialist for sinus issues, but is otherwise healthy. Most recent RCs were to chase up an ENT appointment, and for an injury to his ribs. |
| Alice | Female | 20s | In good health, but experiences disordered eating and periods of low mood. She has had limited contact with her GP over the past five years, but contacted her GP with concerns about her eating and menstrual cycle. She completed an online triage form followed by a telephone RC. |
| Ami | Female | 20s | Generally fit and healthy. Regularly takes iron tablets for anaemia. Five previous experiences of online consultations, mostly for her anaemia |
| Andrew | Male | 30s | Fit and well, with no chronic conditions. Had several RCs with his GP in relation to a whiplash injury from a car accident, an incident when he had potentially eaten some poisonous seeds, and for toothache. |
| Bob | Male | 50s | Has hypertension, and still feels the consequences of injuries to his ribs and leg following a vehicle collision several years ago, including ongoing issues with the venous system in his leg. He had experience of video RCs with his GP. |
| Brian | Male | 20s | Fit and healthy. Regularly takes medication for heartburn. Three previous experiences of online consultations focused on heart burn |
| Carol | Female | 50s | Medical history includes osteoarthritis and degenerative disc disease. She has experience of online triage forms and video RCs with her GP in relation to concerns about bleeding related to bowel movements. |
| Chantelle | Female | 50s | Fit and heathy. Works as a nurse so spends much of her days walking. Over 5 experiences of online consultations due to ongoing knee issues. |

continues ...

... continued

| **Pseudonym** | **Gender** | **Age** | **Biographical sketch** |
| --- | --- | --- | --- |
| David | Male | 20s | Suffers with back pain, but is otherwise fit and well. He has experience of online triage forms and phone RCs with his GP, most recently in relation to discovering a lump in his testicle. |
| Elizabeth | Female | 60s | Experiences chronic back pain due to bulging disks, and has hypothyroidism, but is otherwise healthy and fit. His most recent RC with a GP was related to blood tests to monitor hypothyroidism. He also has two children who regularly see specialists. |
| Faith | Female | 20s | Fit and well: has mild anaemia, but no chronic conditions. Most recent contacts with GP with RCs to arrange cervical screening, and to discuss management of headaches and neck pain following a car accident. |
| Jamal | Male | 60s | Has mild asthma, eczema, hay fever, and chronic back pain. Does not see GP or specialist regularly for anything, and his most recent RC with a GP was prompted by a skin rash. |
| Jamie | Male | 20s | Generally fit and well, and did not elaborate on the reasons for his RCs with GPs, but did mention that has had several RCs. |
| John | Male | 40s | In good health, with no medical conditions or regular medication. Two experiences of RCs, including one on when on vacation |
| Kate | Female | 50s | Has a chronic condition. She had experience of online RCs to discuss new treatments for her diagnosed condition, to investigate another potential chronic condition, and because of a query about tonsillitis. |
| Keith | Male | 60s | Has ulcerative colitis, and because he has not yet had his medications managed effectively, he has had several RCs with his GP about it. He also had on RC for symptoms of shingles. |
| Molly | Female | 30s | Generally in good health. Has had several online RCs with her GP in relation to new rash on her foot, her mental health, and gynaecological concerns. |
